# Risk of Corneal Ulcer in Patients with Diabetes Mellitus: A Retrospective Large-Scale Cohort Study

**DOI:** 10.1038/s41598-020-64489-0

**Published:** 2020-04-30

**Authors:** Yuh-Shin Chang, Ming-Cheng Tai, Chung-Han Ho, Chin-Chen Chu, Jhi-Joung Wang, Sung-Huei Tseng, Ren-Long Jan

**Affiliations:** 1 Department of Ophthalmology, Chi Mei Medical Center, Tainan, Taiwan; 2 Graduate Institute of Medical Science, College of Health Science, Chang Jung Christian University, Tainan, Taiwan; 3 Department of Ophthalmology, Tri-Service General Hospital, National Defense Medical Center, Taipei, Taiwan; 4 Department of Medical Research, Chi Mei Medical Center, Tainan, Taiwan; 5 Department of Hospital and Health Care Administration, Chia Nan University of Pharmacy and Science, Tainan, Taiwan; 6 Department of Anesthesiology, Chi Mei Medical Center, Tainan, Taiwan; 7 Department of Recreation and Health-Care Management, Chia-Nan University of Pharmacy and Science, Tainan, Taiwan; 8 Department of Ophthalmology, National Cheng Kung University Hospital, College of Medicine, National Cheng Kung University, Tainan, Taiwan; 9 Department of Pediatrics, Chi Mei Medical Center, Liouying, Tainan, Taiwan

**Keywords:** Diseases, Endocrinology, Risk factors

## Abstract

This nationwide, retrospective, matched cohort study was designed to investigate the risk of corneal ulcer in patients with diabetes mellitus (DM). It included 238,701 patients with DM, recruited between 2003 and 2005 from the Longitudinal Cohort of Diabetes Patients database. The control group included the same number of age- and sex-matched non-DM patients selected from the Taiwan Longitudinal Health Insurance Database, 2000. The data of each patient were collected from the index date until December 2013. The incidence of corneal ulcer was compared between the two groups. In total, 2,549 patients with DM and 1,988 controls developed corneal ulcer during the follow-up period, resulting in an incidence rate for corneal ulcers that was 1.27 times (95% confidence interval [CI] = 1.20–1.35; P < 0.001) higher in patients with DM than in controls. After adjustment for potential confounders, including hyperlipidemia, hypertension, congestive heart failure, coronary artery disease, and chronic renal disease, patients with DM were 1.31 times (95% CI, 1.24–1.40; P < 0.05) more likely than the cohort to develop corneal ulcers. In conclusion, this study shows that DM increases the risk of corneal ulcer. Therefore, close collaboration between ophthalmologists and endocrinologists is important to ensure timely ophthalmology visits.

## Introduction

The global increase in the prevalence of diabetes mellitus (DM) is an important public health burden related to its accompanying morbidity and mortality^1–3^. The ocular complications resulting from DM are leading causes of blindness and have become general public health problems^4–6^. Although diabetic retinopathy is the most well-known ocular complication, DM may also result in corneal ulceration.

Corneal ulcers caused by infection from pathogens, including bacteria, viruses, and fungi, are a leading cause of visual impairment and blindness worldwide^7–9^. The most frequent clinical presentation of corneal ulcer includes redness, severe pain, photophobia, watering of eyes, pus formation, or blurred visual acuity. Contact lens use is a major risk factor and the most common aetiology for the pathogenesis of corneal ulcers^10–12^. It may also occur because of dry eyes resulting from an unbalanced and unstable tear film, compromised ocular surface associated with corneal epithelium abnormality, and ocular surface injury associated with decreased corneal sensitivity^7–9^.

The reduction in the secretion and stability of the precorneal tear film may be a result of the decreased trophic effect of the trigeminal sensory nerves on the cornea in patients with DM^13,14^. Compromised ocular surface is common in patients with DM and leads to increased prevalence of epithelial fragility, punctate keratopathy, and persistent epithelial defects^15–17^. Decreased corneal sensitivity and abnormal neural regulation is known to result in delayed epithelial wound healing and increased risk of corneal ulcer following corneal trauma in patients with DM^18^. Considering the common pathogenic mechanisms in DM and corneal ulcer, it is important to determine whether DM is a predictor of corneal ulcer.

Several previous studies have discussed the association between DM and corneal ulcer, but they were limited by the small number of patients or the absence of comparative control data^19–21^. Therefore, this study used a nationwide population-based dataset to design a cohort study for assessing the association between DM and risk of corneal ulcer in Taiwan.

## Materials and Methods

### Database

The data for the cohort study were obtained from the Taiwan National Health Insurance Research Database (NHIRD), which includes the International Classification of Diseases, Ninth Revision, Clinical Modification (ICD-9-CM) diagnoses, prescriptions details, procedure codes, and expenses. NHIRD also provides details regarding encrypted patient identification, age, gender, birthday, and dates of admission and discharge. The Institutional Review Board of Chi Mei Medical Center exempted the review of the study because no identifiable personal information could be analysed and the informed consent was waived. In this study, all procedures were performed in accordance with the ethical standards of the institutional research committee and with the latest version of the declaration of Helsinki.

### Selection of patients and variables

This retrospective cohort study included two groups: a new-onset DM group and a matched non-DM (control) group; participants of both groups were recruited between 2003–2005. In total, 238,701 DM patients (code 250) were diagnosed between January 1, 2003 and December 31, 2005. These new-onset DM patients were chosen from the Longitudinal Cohort of Diabetes Patients Database, which is a subdivision data of the NHIRD and it has a random sample of 120,000 DM patients per year from 1996 to 2013. The database was released by Taiwan National Health Research Institutes and applied by the public through formal application. Many research studies used the same database and later had many publications^22–24^. Patients with missing data, age less than 20 years, and those being diagnosed with corneal ulcer before DM were excluded. A diagnosis of corneal ulcer was defined as conditions diagnosed under the International Classification of Diseases, Ninth Revision, Clinical Modification (ICD-9-CM) code 370.0, including 370.01 (marginal corneal ulcer), 370.02 (ring corneal ulcer), 370.03 (central corneal ulcer), 370.04 (hypopyon ulcer), 370.05 (mycotic corneal ulcer), and 370.06 (perforated corneal ulcer), but not 370.07 (Mooren’s ulcer).

For each DM patient, one non-DM control was selected from a sub-data of the NHIRD, Longitudinal Health Insurance Database, 2000. This data includes the information of 1 million beneficiaries (4.34% of the total population) randomly selected in 2000. In total, 238,701 non-DM controls were matched to DM patients by age, gender and index date. The index date for DM patients was the date of initial diagnosis. Controls diagnosed with DM or corneal ulcer before the index date were excluded. To determine the incidence of corneal ulcer, each patient was tracked until death or the end of year 2013, whichever was earlier.

To discriminate patients who developed corneal ulcer following DM, each patient with demographic data was traced from index date until December 2013. The data about the comorbidities of patients, including hyperlipidemia (code 272), hypertension (codes 401–405), coronary artery disease (codes 410–414), congestive heart failure (code 428), and chronic renal diseases (codes 582–588 except 587 and 584) were also gathered. The inclusion criterion for hyperlipidemia, hypertension, coronary artery disease, congestive heart failure, and chronic renal disease was as follows: medical record at least once in hospitalization or ≥3 times in outpatient visits within 1 year before the index date.

### Statistical analysis

SAS 9.4 for Windows (SAS Institute, Inc., Cary, NC, USA) was used for statistical analyses. Pearson’s chi-square tests were employed to compare the demographics and comorbidities between the DM and control groups. The incidence of corneal ulcer was determined as the patient quantity with corneal ulcer identified during the tracking period divided by the sum of person-years (PY) for each group by age, gender, and relevant comorbidities. The incidence rate ratio (IRR), which compared the corneal ulcer risk between the DM and control groups, was estimated using Poisson regression analysis. The cumulative incidence rates for corneal ulcers and their differences were calculated and analyzed by Kaplan–Meier analyses and log-rank tests, respectively. Cox proportional hazards regression was employed to calculate the adjusted hazard ratios (HRs) for the development of corneal ulcer. The data are exhibited as means and their standard deviations (SDs), with 95% confidence intervals (CIs) where applicable. Statistical significance was defined as P < 0.05. Similar statistical analysis and method description were used in our previous study^22–24^.

## Results

### Demographic data

Between 2003 and 2005, 238,701 patients with DM and non-DM 238,701 controls were enrolled after eliminating unqualified subjects. Table 1 reveals the demographics for DM patients and matched controls. The mean age of the DM patients and controls was 55.11 (SD, 14.83) years. Of the 238,701 patients with DM, 132,772 (55.62%) were men and 105,929 (44.38%) women; further, of the 238,701 patients, 90,568 (37.94%) were aged 20–50 years, 85,453 (35.80%) were aged 50–64 years, and 62,680 (26.26%) were aged ≥65 years. The DM patients group exhibited a remarkably higher prevalence of comorbidities, including hyperlipidemia, hypertenstion, coronary artery disease, congestive heart failure, and chronic renal disease, than did the control group.

**Table 1:** Demographic characteristics and comparison of comorbid disorders between the diabetes mellitus (DM) and control groups.

	DM N = 238701	Control N = 238701	P-value
Age at index date (years; mean ± SD)	55.11 ± 14.83	55.11 ± 14.83	1.0000
**Age at index date (years)**			
20–50	90568 (37.94)	90568 (37.94)	1.0000
50–64	85453 (35.80)	85453 (35.80)	
≥65	62680 (26.26)	62680 (26.26)	
**Sex**			
Male	132772 (55.62)	132772 (55.62)	1.0000
Female	105929 (44.38)	105929 (44.38)	
**Baseline comorbidities**			
HTN	73890 (30.96)	26089 (10.93)	<0.0001
HPL	24722 (10.36)	5952 (2.49)	<0.0001
CHF	6411 (2.69)	1780 (0.75)	<0.0001
CAD	21418 (8.97)	7683 (3.22)	<0.0001
CRD	5912 (2.48)	2215 (0.93)	<0.0001

Note: Demographic characteristics and comorbid disorders were compared between the DM and control groups by using Pearson’s chi-square tests. Abbreviations: HTN, hypertension; HPL, hyperlipidemia; CHF, congestive heart failure; CAD, coronary heart disease; CRD, chronic renal disease

### Incidence rates for corneal ulcer

During the tracking period, 4,537 (4,537/477,402, 0.950%) patients developed corneal ulcer, with the proportion being significantly higher in the DM group (2,549/238,701, 1.068%) than in the control group (1,988/238,701, 0.833%; Table 2). In addition, a remarkable intergroup difference was observed in the corneal ulcer incidence rate (DM, 12.19/10000 PY; control, 9.57/10000 PY) and IRR (1.27, 95% CI = 1.20–1.35, P < 0.0001; Table 2).

**Table 2:** Risk of corneal ulcer in the diabetes mellitus (DM) and control groups.

	DM				Control				IRR (95% CI)	P-value
	N	Corneal ulcer	PY	Incidence rate^a^	N	Corneal ulcer	PY	Incidence rate^a^		
All	238701	2549	2090353	12.19	238701	1988	2078030	9.57	1.27 (1.20-1.35)	<0.0001
**Age at index date (years)**										
20–50	90568	1102	832987	13.23	90568	864	843326	10.25	1.29 (1.18-1.41)	<0.0001
50–64	85453	862	768153	11.22	85453	732	763355	9.59	1.17 (1.06-1.29)	0.0018
≥65	62680	585	489212	11.96	62690	392	471349	8.32	1.44 (1.27-1.63)	<0.0001
**Sex**										
Male	132772	1500	1144252	13.11	132772	1142	1136159	10.05	1.30 (1.21-1.41)	<0.0001
Female	105929	1049	946101	11.09	105929	846	941871	8.98	1.23 (1.13-1.35)	<0.0001
**Baseline comorbidities**										
HTN	73890	686	622764	11.02	26089	200	223190	8.96	1.23 (1.05-1.44)	0.0102
HPL	24722	238	219681	10.83	5952	59	52681	11.20	0.97 (0.73-1.29)	0.8195
CHF	6411	47	45760	10.27	1780	12	13284	9.03	1.14 (0.60-2.14)	0.6915
CAD	21418	188	174828	10.75	7683	66	64325	10.26	1.05 (0.79-1.39)	0.7429
CRD	5912	48	41102	11.68	2215	16	15401	10.9	1.12 (0.64-1.98)	0.6854

Note: A Poisson regression analysis was performed to calculate the incidence rate ratio.

Abbreviations: PY, person-years; IRR, incidence rate ratio; CI, confidence interval; HTN, hypertension; HPL, hyperlipidemia; CHF, congestive heart failure; CAD, coronary heart disease; CRD, chronic renal disease.

^a^Incidence rate: per 10,000 person-years.

DM patients aged 20–50 years exhibited the highest corneal ulcer incidence (13.23/10000 PY), followed by those aged ≥65 years (11.96/10000 PY) and 50–64 years (11.22/10000 PY). The values of IRR were significantly higher for the three different DM age groups than for the age-matched control groups (Table 2).

The corneal ulcer incidence rate was 13.11/10000 PY for male DM patients and 10.05/10000 PY for male controls (IRR = 1.30; 95% CI = 1.21–1.41; P < 0.0001). A significant difference was also observed between female DM patients and female controls (IRR = 1.23; 95% CI = 1.13–1.35; P < 0.0001; Table 2).

**Figure 1:**
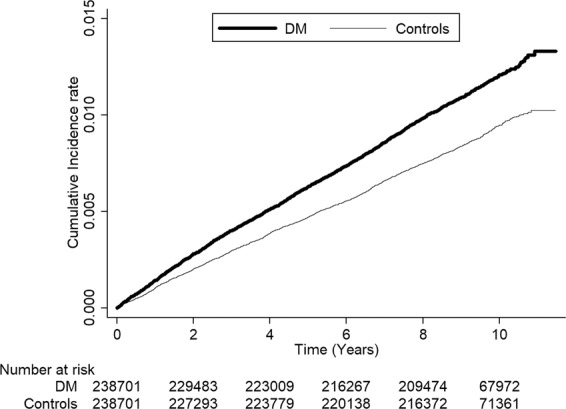
Cumulative incidence of corneal ulcer in patients with diabetes mellitus (DM) and controls during the follow-up period.

In the DM patients, the corneal ulcer incidence rates decreased in the following order according to the comorbidities: chronic renal disease (11.68/10000 PY), hypertension (11.02/10000 PY), hyperlipidemia (10.83/10000 PY), coronary artery disease (10.75/10000 PY), and congestive heart failure (10.27/10000 PY). In particular, the incidence was 1.23 times higher in patients with DM and hypertension than in age-matched controls (IRR = 1.23; 95% CI = 1.05–1.44; P = 0.0102). The IRR for corneal ulcer in DM patients with other comorbidities did not indicate a statistically greater risk than in the corresponding controls. Additionally, by using Kaplan–Meier analyses, DM group showed higher cumulative incidence rate of corneal ulcer than the control group; the log-rank test findings were also significant (P < 0.0001; Fig. 1).

### Hazard ratios for corneal ulcers

Table 3 reveals the crude and adjusted HRs for corneal ulcer during the tracking period. After adjustment for age, sex, and the selected comorbidities, DM remained an independent risk for corneal ulcer (adjusted HR = 1.31; 95% CI = 1.24–1.40). In both groups, patients aged between 20−50 years (adjusted HR, 1.15; 95% CI = 1.06–1.25; P < 0.05) and of the male sex (adjusted HR, 1.14; 95% CI = 1.08–1.21; P < 0.05) were at higher risk for the development of corneal ulcer. All selected comorbidities were not independent risks for corneal ulcer.

**Table 3:** Crude and adjusted hazard ratios and 95% confidence intervals (CI) from Cox proportional hazard regression analysis for corneal ulcer during the follow-up period in the study cohort.

	Crude hazard ratio (95% CI)	Adjusted hazard ratio (95% CI)
**DM**		
Yes	1.29* (1.22-1.37)	1.31* (1.24-1.40)
No	1.00	1.00
**Age at index date**		
20–50	1.18* (1.10-1.28)	1.15* (1.06-1.25)
50–64	1.05 (0.97-1.13)	1.03 (0.95-1.12)
≥65	1.00	1.00
**Sex**		
Male	1.15* (1.08-1.22)	1.14* (1.08-1.21)
Female	1.00	1.00
**Baseline comorbidities**		
HTN		
Yes	0.96 (0.89-1.03)	0.93 (0.86-1.01)
No	1.00	1.00
**HPL**		
Yes	1.01 (0.90-1.14)	0.97 (0.85-1.09)
No	1.00	1.00
**CHF**		
Yes	0.92 (0.71-1.19)	0.92 (0.71-1.20)
No	1.00	1.00
**CAD**		
Yes	0.98 (0.86-1.11)	1.00 (0.88-1.15)
No	1.00	1.00
**CRD**		
Yes	1.04 (0.81-1.33)	1.04 (0.81-1.34)
No	1.00	1.00

Note: The adjusted hazard ratio for developing corneal ulcer was calculated using a Cox proportional hazard regression analysis.

Abbreviations: DM, diabetes mellitus; HTN, hypertension; HPL, hyperlipidemia; CHF, congestive heart failure; CAD, coronary heart disease; CRD, chronic renal disease.

*P-value <0.05.

## Discussion

This study is the largest population-based study that has been conducted to explore the relationship between DM and subsequent corneal ulcer. The authors analyzed 238,701 patients with DM and 238,701 control subjects. The incidence rate of corneal ulcer in patients with DM was found to be 1.27 times higher than that in controls, and the relative risk of corneal ulcer in these patients was 1.31 times higher than that in the total cohort, after adjustment for age, sex, and relevant comorbidities.

Several studies have reported the occurrence of DM in patients with infectious keratitis. Badawi *et al*. studied the prevalence of DM in 245 patients with corneal ulcer and suggested that DM was a predisposing factor (present in 15.1% of the patients)^21^. Inoue *et al*. screened for DM in 30 culture-proven cases of corneal ulcer due to *Moraxella* infection and found that the occurrence of DM was 23.8% in these patients; hence, in line with earlier study findings, they reported that DM was the predominant systemic predisposing factor for *Moraxella* keratitis^19–21^. These studies were limited because of their small sample size; in contrast, the present study is the largest nationwide, population-based cohort study to investigate the risk of corneal ulcer following DM in Taiwan.

The possible associated pathogenic mechanisms for corneal ulcer in patients with DM include dryness of eye, compromised ocular surface, and decreased corneal sensitivity. Dry eye symptoms due to reduced stability, secretion, and quality of the precorneal tear film are common in patients with DM. Dryness of the eye in patients with DM render them at greater risk of compromised ocular surface, including superficial punctate keratitis, recurrent corneal erosions, and persistent epithelial defects^18,25^. In addition, several studies found that the density of the corneal sub-basal nerve plexus, which is responsible for maintaining corneal sensitivity and normal epithelial metabolism, is reduced in patients with DM^26,27^. Once a patient with DM develops dry eye, compromised ocular surface, and reduced corneal sensitivity, the vulnerability to mild trauma, which is the leading cause of corneal ulcer, increases. These abnormalities in patients with DM play an important role in corneal ulcer development.

In this study, male patients were at a significant higher risk of developing corneal ulcer, after accounting for age and comorbidities in both groups (Table 3). The observation of male predominance is found in several reports^21,28–30^. The male predominance in corneal ulcer could be attributed to the greater extent of outdoor activities among men. This likely makes them more prone to corneal injury by external agents^21,28,31^.

The study results show that patients aged between 20–50 years showed a significantly greater incidence of corneal ulcer development in the cohort (Table 3). A similar observation was made in earlier studies^21,28,31^. This finding could be explained by the fact that younger individuals are more likely to engage in rigorous physical activities or wear contact lenses, thus increasing the risk of ocular surface trauma and subsequent corneal infection.

This study has several merits. These include a high statistical power and accuracy of risk assessment is in our nationwide, population-based study, because a large sample size of DM patients was included in our dataset. Additionally, because patients with visual disturbances visit ophthalmologists rather than general practitioners in Taiwan, selection bias in referral centers and chances of misdiagnosis were low in this study. Finally, this study has a strong evidence level because this cohort study assessed the incidence of corneal ulcer in the DM and control groups with a maximum longitudinal data of 10 years.

This study also has some limitations. Because the medical histories of the sampled patients could only be traced back to 1996, a history of DM before January 1996 could not be confirmed. The influence of blood sugar control on the risk of developing corneal ulcer could not be evaluated, because the insurance claims data did not include information on the hemoglobin A_1C_ level or the current blood sugar value. Several important confounding factors including occupation, contact lens use, plant or mud exposure, and mild ocular trauma could not be evaluated. It is likely that significantly less patients with diabetes used contact lenses owing to the general belief that wearing contact lenses is contradicted in diabetics. If this were true, the current study might actually be underestimating the risk of keratitis in patients with diabetes. This is a retrospective study, and therefore, the results may be less robust in establishing an epidemiological risk or a clear definition of keratitis. Finally, the diagnosis of DM, corneal ulcer, and other comorbid disorders were based on the ICD-9-CM codes, which may lead to disease misclassification.

In summary, this study shows that the risk of corneal ulcer is higher in patients with DM than in those without DM. DM was identified as an independent risk after adjusting for relevant comorbidities in the total cohort. Although the study reports a 1.31-fold increased risk of keratitis in patients with diabetes, which is of minor clinical importance, it is an important finding from a pathophysiological perspective, and is consistent with known compromise of the ocular surface in patients with diabetes.
